# 3-[Bis(*p*-tolyl­sulfon­yl)amino]-*N*-(4-chloro­benz­yl)-6-(3,4-dichloro­phen­yl)thieno[2,3-*b*]pyridine-2-carboxamide

**DOI:** 10.1107/S160053681100290X

**Published:** 2011-01-26

**Authors:** Hai-Yun He, Hong-Ze Li, Li Yang

**Affiliations:** a State Key Laboratory of Biotherapy and Cancer Center, West China Hospital, West China Medical School, Sichuan University, Chengdu 610041, People’s Republic of China; b West China School of Pharmacy, Sichuan University, Chengdu 610041, People’s Republic of China

## Abstract

In the title compound, C_35_H_26_Cl_3_N_3_O_5_S_3_, the dihedral angle between the mean plane through the thieno[2,3-*b*]pyridine ring system and the attached benzene ring is 3.89 (6)°. The mol­ecular conformation is stabilized by an intra­molecular N—H⋯O hydrogen bond. In the crystal, mol­ecules are linked by inter­molecular C—H⋯O hydrogen bonds, forming chains parallel to [100].

## Related literature

For general background to the biological properties of thieno[2,3-*b*]pyridine derivatives, see: Litvinov *et al.* (2005 ▶).
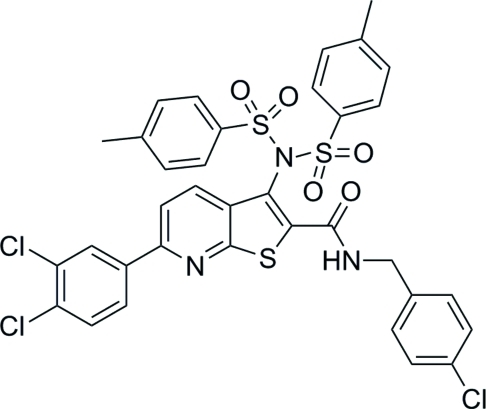


## Experimental

### 

#### Crystal data

C_35_H_26_Cl_3_N_3_O_5_S_3_
*M* *_r_* = 771.15
Monoclinic, 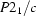

*a* = 25.8238 (8) Å
*b* = 9.1634 (2) Å
*c* = 14.8366 (5) Å
β = 102.314 (3)°
*V* = 3430.07 (17) Å^3^
*Z* = 4
Mo *K*α radiation
μ = 0.50 mm^−1^
*T* = 294 K
0.36 × 0.28 × 0.16 mm

#### Data collection

Oxford Diffraction Xcalibur Eos diffractometer
Absorption correction: multi-scan (*CrysAlis PRO*; Oxford Diffraction, 2010 ▶) *T*_min_ = 0.695, *T*_max_ = 1.0
14766 measured reflections
7014 independent reflections
4515 reflections with *I* > 2σ(*I*)
*R*_int_ = 0.024

#### Refinement

*R*[*F* ^2^ > 2σ(*F* ^2^)] = 0.043
*wR*(*F* ^2^) = 0.105
*S* = 1.03
7014 reflections
444 parameters
H-atom parameters constrained
Δρ_max_ = 0.28 e Å^−3^
Δρ_min_ = −0.35 e Å^−3^

### 

Data collection: *CrysAlis PRO* (Oxford Diffraction, 2010 ▶); cell refinement: *CrysAlis PRO*; data reduction: *CrysAlis PRO*; program(s) used to solve structure: *SHELXS97* (Sheldrick, 2008 ▶); program(s) used to refine structure: *SHELXL97* (Sheldrick, 2008 ▶); molecular graphics: *OLEX2* (Dolomanov *et al.*, 2009 ▶); software used to prepare material for publication: *SHELXTL* (Sheldrick, 2008 ▶) and *OLEX2*.

## Supplementary Material

Crystal structure: contains datablocks global, I. DOI: 10.1107/S160053681100290X/rz2547sup1.cif

Structure factors: contains datablocks I. DOI: 10.1107/S160053681100290X/rz2547Isup2.hkl

Additional supplementary materials:  crystallographic information; 3D view; checkCIF report

## Figures and Tables

**Table 1:** Hydrogen-bond geometry (Å, °)

*D*—H⋯*A*	*D*—H	H⋯*A*	*D*⋯*A*	*D*—H⋯*A*
N3—H3⋯O2	0.86	2.11	2.945 (3)	162
C34—H34⋯S1^i^	0.93	2.79	3.714 (2)	172

Symmetry code: (i) 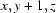.

**Fig. 1.**
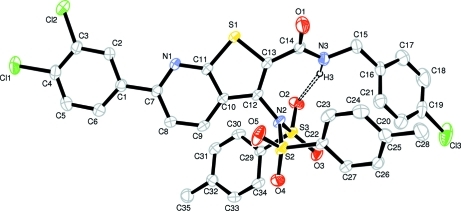
The molecular structure of the title compound, with displacement ellipsoids drawn at the 30% probability level. The intramolecular N—H···O hydrogen bond is shown as a dashed line.

**Fig. 2.**
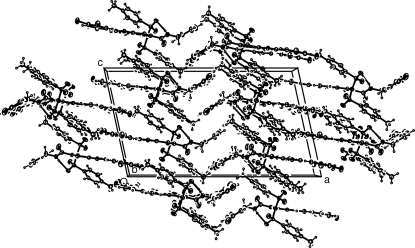
Packing diagram of the title compound viewed along the b axis.

## supplementary crystallographic information

### Comment

Thieno[2,3-*b*]pyridine derivatives are of great importance owing to their wide biological properties (Litvinov *et al.*, 2005). The title compound is one of the key intermediates in our synthetic investigations of anticancer drugs. We report here its crystal structure.

In the tile compound (Fig. 1), the thieno[2,3-*b*]pyridine ring system is approximately planar (maximum deviation 0.048 (2) Å for atom C8) and forms a dihedral angle of 3.89 (6)° with the attached benzene ring. The molecular conformation is stabilized by an intramolecular N—H···O hydrogen bond (Table 1). In the crystal structure (Fig. 2), the molecules are linked into chains parallel to the [100] direction by C···H···O hydrogen bonds.

### Experimental

To a solution of 3-amino-*N*-(4-chlorobenzyl)-6-(3,4-dichlorophenyl)thieno[2,3-*b*]pyridine-2-carboxamide (1.39 g, 3 mmol) in tetrahydrofuran (10 ml) and triethylamine (0.63 ml) was added *p*-toluenesulfonyl chloride (1.14 g, 6 mmol). After stirring for 5 minutes, *N*,*N*-4-dimethylaminopyridine (48 mg, 0.39 mmol) was added and the mixture was stirred at room temperature until the reaction was completed (as monitored by TLC). The reaction mixture was concentrated *in vacuo* and the resulting residue was treated with a dilute HCl solution and extracted with ethyl acetate. The organic phase was dried over MgSO_4_ and then was evaporated under reduced pressure. The residue was chromatographed on silica gel using petroleum ether-ethyl acetate (9:1 *v*/*v*) as eluent. The product was recrystallized from ethanol to afford the title compound as an off-white solid (yield: 60%). Crystals suitable for X-ray analysis were obtained by slow evaporation of an ethyl acetate solution.

### Refinement

All H atoms were positioned geometrically (N—H = 0.86 Å; C—H = 0.93–0.97 Å) and refined using a riding model, with *U*_iso_(H) = 1.2*U*_eq_(C, N) or 1.2*U*_eq_(C) for methyl H atoms. The crystal was ground into a spheroidal shape.

### Crystal data

**Table:**

C_35_H_26_Cl_3_N_3_O_5_S_3_	*F*(000) = 1584
*M**_r_* = 771.15	*D*_x_ = 1.493 Mg m^−^^3^
Monoclinic, *P*2_1_/*c*	Mo *K*α radiation, λ = 0.7107 Å
Hall symbol: -P 2ybc	Cell parameters from 6766 reflections
*a* = 25.8238 (8) Å	θ = 3.1–29.1°
*b* = 9.1634 (2) Å	µ = 0.50 mm^−^^1^
*c* = 14.8366 (5) Å	*T* = 294 K
β = 102.314 (3)°	Block, colourless
*V* = 3430.07 (17) Å^3^	0.36 × 0.28 × 0.16 mm
*Z* = 4	

### Data collection

**Table:**

Oxford Diffraction Xcalibur Eos diffractometer	7014 independent reflections
Radiation source: fine-focus sealed tube	4515 reflections with *I* > 2σ(*I*)
graphite	*R*_int_ = 0.024
Detector resolution: 16.0874 pixels mm^-1^	θ_max_ = 26.4°, θ_min_ = 3.1°
ω scans	*h* = −28→32
Absorption correction: multi-scan (*CrysAlis PRO*; Oxford Diffraction, 2010)	*k* = −10→11
*T*_min_ = 0.695, *T*_max_ = 1.0	*l* = −18→9
14766 measured reflections	

### Refinement

**Table:**

Refinement on *F*^2^	Primary atom site location: structure-invariant direct methods
Least-squares matrix: full	Secondary atom site location: difference Fourier map
*R*[*F*^2^ > 2σ(*F*^2^)] = 0.043	Hydrogen site location: inferred from neighbouring sites
*wR*(*F*^2^) = 0.105	H-atom parameters constrained
*S* = 1.03	*w* = 1/[σ^2^(*F*_o_^2^) + (0.0514*P*)^2^] where *P* = (*F*_o_^2^ + 2*F*_c_^2^)/3
7014 reflections	(Δ/σ)_max_ = 0.001
444 parameters	Δρ_max_ = 0.28 e Å^−^^3^
0 restraints	Δρ_min_ = −0.35 e Å^−^^3^

### Special details

**Table:**

Geometry. All e.s.d.'s (except the e.s.d. in the dihedral angle between two l.s. planes) are estimated using the full covariance matrix. The cell e.s.d.'s are taken into account individually in the estimation of e.s.d.'s in distances, angles and torsion angles; correlations between e.s.d.'s in cell parameters are only used when they are defined by crystal symmetry. An approximate (isotropic) treatment of cell e.s.d.'s is used for estimating e.s.d.'s involving l.s. planes.
Refinement. Refinement of *F*^2^ against ALL reflections. The weighted *R*-factor *wR* and goodness of fit *S* are based on *F*^2^, conventional *R*-factors *R* are based on *F*, with *F* set to zero for negative *F*^2^. The threshold expression of *F*^2^ > σ(*F*^2^) is used only for calculating *R*-factors(gt) *etc*. and is not relevant to the choice of reflections for refinement. *R*-factors based on *F*^2^ are statistically about twice as large as those based on *F*, and *R*- factors based on ALL data will be even larger.

### Fractional atomic coordinates and isotropic or equivalent isotropic displacement parameters (Å^2^)

**Table:**

	*x*	*y*	*z*	*U*_iso_*/*U*_eq_	
S3	0.28910 (2)	0.51376 (6)	0.40412 (4)	0.04300 (16)	
S2	0.23044 (2)	0.51746 (6)	0.20959 (4)	0.04806 (17)	
S1	0.24607 (2)	−0.00731 (5)	0.31174 (4)	0.04490 (16)	
Cl2	0.02235 (3)	−0.41937 (7)	0.38236 (6)	0.0764 (2)	
C12	0.24509 (9)	0.2738 (2)	0.31359 (14)	0.0375 (5)	
Cl3	0.52839 (4)	0.81264 (11)	0.41121 (7)	0.1161 (4)	
N2	0.26006 (7)	0.42373 (17)	0.30696 (12)	0.0404 (5)	
O3	0.30905 (7)	0.64478 (16)	0.37327 (12)	0.0568 (5)	
C2	0.05859 (9)	−0.1498 (2)	0.35900 (16)	0.0453 (6)	
H2	0.0887	−0.1967	0.3487	0.054*	
O4	0.21021 (7)	0.64976 (17)	0.23812 (13)	0.0641 (5)	
N1	0.14607 (7)	0.00580 (17)	0.33823 (12)	0.0400 (4)	
C4	−0.02829 (10)	−0.1637 (3)	0.39124 (18)	0.0542 (7)	
C9	0.14909 (10)	0.3121 (2)	0.33334 (16)	0.0448 (6)	
H9	0.1497	0.4136	0.3323	0.054*	
N3	0.36706 (7)	0.2419 (2)	0.31819 (14)	0.0500 (5)	
H3	0.3600	0.3039	0.3574	0.060*	
C30	0.22700 (10)	0.4507 (2)	0.52574 (16)	0.0480 (6)	
H30	0.2448	0.3620	0.5346	0.058*	
C33	0.17583 (10)	0.7139 (2)	0.50220 (17)	0.0521 (6)	
H33	0.1590	0.8042	0.4949	0.062*	
C8	0.10435 (9)	0.2384 (2)	0.34211 (16)	0.0459 (6)	
H8	0.0740	0.2904	0.3461	0.055*	
O2	0.32449 (6)	0.40866 (16)	0.45588 (11)	0.0555 (5)	
C14	0.33000 (9)	0.1470 (2)	0.27949 (17)	0.0445 (6)	
C1	0.05624 (9)	0.0017 (2)	0.35697 (15)	0.0428 (5)	
C26	0.34030 (9)	0.7161 (3)	0.10029 (17)	0.0492 (6)	
H26	0.3548	0.8089	0.0996	0.059*	
C13	0.27713 (9)	0.1581 (2)	0.30390 (15)	0.0385 (5)	
C31	0.18809 (10)	0.4803 (2)	0.57265 (16)	0.0485 (6)	
H31	0.1794	0.4105	0.6125	0.058*	
C34	0.21404 (10)	0.6856 (2)	0.45385 (18)	0.0513 (6)	
H34	0.2226	0.7549	0.4137	0.062*	
C23	0.29804 (11)	0.4418 (3)	0.10271 (18)	0.0560 (7)	
H23	0.2839	0.3487	0.1039	0.067*	
O5	0.19592 (7)	0.41295 (19)	0.15714 (12)	0.0723 (6)	
C25	0.35659 (10)	0.6059 (3)	0.04872 (17)	0.0524 (6)	
O1	0.33772 (7)	0.0519 (2)	0.22660 (15)	0.0826 (6)	
C6	0.00994 (10)	0.0669 (3)	0.3697 (2)	0.0625 (7)	
H6	0.0070	0.1681	0.3672	0.075*	
C15	0.41940 (9)	0.2435 (3)	0.2959 (2)	0.0628 (7)	
H15A	0.4158	0.2275	0.2303	0.075*	
H15B	0.4406	0.1646	0.3284	0.075*	
C16	0.44723 (10)	0.3864 (3)	0.32238 (18)	0.0513 (6)	
C20	0.44696 (12)	0.6484 (3)	0.3235 (2)	0.0670 (8)	
H20	0.4294	0.7360	0.3067	0.080*	
C24	0.33542 (11)	0.4684 (3)	0.05170 (18)	0.0589 (7)	
H24	0.3467	0.3927	0.0187	0.071*	
C28	0.39610 (11)	0.6335 (3)	−0.0109 (2)	0.0760 (9)	
H28C	0.4306	0.6018	0.0210	0.114*	
H28B	0.3971	0.7360	−0.0239	0.114*	
H28A	0.3858	0.5804	−0.0676	0.114*	
C21	0.42232 (11)	0.5173 (3)	0.29841 (19)	0.0616 (7)	
H21	0.3877	0.5173	0.2641	0.074*	
C5	−0.03178 (10)	−0.0148 (3)	0.3858 (2)	0.0700 (8)	
H5	−0.0627	0.0316	0.3932	0.084*	
C17	0.49892 (11)	0.3892 (3)	0.3706 (2)	0.0808 (10)	
H17	0.5169	0.3019	0.3864	0.097*	
C18	0.52456 (13)	0.5207 (4)	0.3958 (3)	0.0962 (11)	
H18	0.5597	0.5219	0.4277	0.115*	
C27	0.30312 (9)	0.6910 (2)	0.15244 (16)	0.0449 (6)	
H27	0.2928	0.7657	0.1872	0.054*	
C19	0.49776 (13)	0.6474 (3)	0.3735 (2)	0.0696 (9)	
C7	0.10363 (9)	0.0855 (2)	0.34518 (15)	0.0393 (5)	
C29	0.23995 (8)	0.5521 (2)	0.46522 (15)	0.0394 (5)	
C32	0.16117 (9)	0.6127 (2)	0.56199 (16)	0.0458 (6)	
C10	0.19388 (9)	0.2313 (2)	0.32598 (14)	0.0354 (5)	
C11	0.18908 (9)	0.0798 (2)	0.32815 (15)	0.0375 (5)	
C35	0.11862 (10)	0.6470 (3)	0.61305 (19)	0.0618 (7)	
H35B	0.1118	0.5625	0.6470	0.093*	
H35A	0.0868	0.6743	0.5699	0.093*	
H35C	0.1299	0.7262	0.6550	0.093*	
C22	0.28132 (9)	0.5534 (2)	0.15253 (15)	0.0408 (5)	
C3	0.01687 (9)	−0.2314 (2)	0.37608 (16)	0.0471 (6)	
Cl1	−0.07975 (3)	−0.26085 (8)	0.41925 (6)	0.0810 (3)	

### Atomic displacement parameters (Å^2^)

**Table:**

	*U*^11^	*U*^22^	*U*^33^	*U*^12^	*U*^13^	*U*^23^
S3	0.0431 (3)	0.0354 (3)	0.0480 (4)	−0.0049 (3)	0.0041 (3)	−0.0046 (3)
S2	0.0455 (4)	0.0443 (3)	0.0499 (4)	−0.0108 (3)	0.0002 (3)	0.0158 (3)
S1	0.0478 (4)	0.0284 (3)	0.0617 (4)	−0.0050 (2)	0.0189 (3)	0.0021 (3)
Cl2	0.0704 (5)	0.0453 (4)	0.1153 (7)	−0.0173 (3)	0.0239 (5)	0.0019 (4)
C12	0.0464 (13)	0.0285 (10)	0.0370 (13)	−0.0075 (10)	0.0079 (11)	0.0037 (9)
Cl3	0.1143 (8)	0.1157 (7)	0.1354 (9)	−0.0746 (6)	0.0649 (7)	−0.0587 (6)
N2	0.0477 (11)	0.0267 (9)	0.0432 (11)	−0.0106 (8)	0.0017 (9)	0.0052 (8)
O3	0.0590 (11)	0.0400 (9)	0.0748 (13)	−0.0212 (8)	0.0220 (10)	−0.0131 (8)
C2	0.0381 (13)	0.0454 (13)	0.0523 (16)	−0.0039 (10)	0.0097 (12)	−0.0011 (11)
O4	0.0583 (11)	0.0537 (10)	0.0838 (14)	0.0138 (8)	0.0228 (10)	0.0298 (9)
N1	0.0413 (10)	0.0343 (9)	0.0460 (11)	−0.0070 (8)	0.0129 (9)	0.0044 (8)
C4	0.0434 (15)	0.0596 (16)	0.0596 (18)	−0.0126 (12)	0.0109 (13)	0.0047 (13)
C9	0.0558 (15)	0.0271 (11)	0.0521 (15)	−0.0044 (11)	0.0126 (12)	0.0032 (10)
N3	0.0449 (12)	0.0478 (11)	0.0613 (14)	−0.0082 (9)	0.0199 (10)	−0.0094 (10)
C30	0.0597 (16)	0.0328 (12)	0.0481 (15)	0.0053 (11)	0.0039 (13)	0.0067 (11)
C33	0.0560 (16)	0.0377 (13)	0.0620 (17)	0.0089 (11)	0.0115 (14)	0.0041 (12)
C8	0.0450 (14)	0.0365 (12)	0.0568 (16)	0.0024 (10)	0.0121 (12)	0.0039 (11)
O2	0.0518 (11)	0.0597 (10)	0.0488 (11)	0.0126 (8)	−0.0028 (8)	−0.0083 (8)
C14	0.0501 (15)	0.0350 (12)	0.0511 (15)	−0.0064 (11)	0.0168 (12)	−0.0010 (11)
C1	0.0418 (13)	0.0450 (13)	0.0417 (13)	−0.0036 (10)	0.0094 (11)	0.0049 (11)
C26	0.0481 (15)	0.0429 (13)	0.0552 (16)	−0.0057 (11)	0.0077 (13)	0.0104 (12)
C13	0.0448 (13)	0.0308 (11)	0.0405 (13)	−0.0082 (10)	0.0102 (11)	0.0016 (9)
C31	0.0591 (16)	0.0416 (13)	0.0430 (14)	−0.0070 (12)	0.0072 (12)	0.0060 (11)
C34	0.0639 (17)	0.0335 (12)	0.0572 (17)	−0.0011 (11)	0.0144 (14)	0.0097 (11)
C23	0.0736 (19)	0.0385 (13)	0.0511 (16)	−0.0112 (13)	0.0029 (14)	−0.0021 (12)
O5	0.0691 (12)	0.0808 (12)	0.0542 (12)	−0.0417 (10)	−0.0155 (10)	0.0201 (9)
C25	0.0463 (15)	0.0660 (17)	0.0419 (15)	0.0071 (12)	0.0027 (12)	0.0103 (13)
O1	0.0674 (13)	0.0762 (13)	0.1151 (18)	−0.0227 (10)	0.0441 (12)	−0.0461 (12)
C6	0.0542 (16)	0.0484 (14)	0.090 (2)	0.0004 (13)	0.0270 (15)	0.0093 (14)
C15	0.0445 (15)	0.0594 (16)	0.090 (2)	−0.0071 (12)	0.0275 (15)	−0.0076 (15)
C16	0.0396 (14)	0.0616 (16)	0.0559 (17)	−0.0103 (12)	0.0171 (13)	−0.0042 (13)
C20	0.071 (2)	0.0626 (17)	0.071 (2)	−0.0108 (15)	0.0216 (17)	−0.0036 (14)
C24	0.0744 (19)	0.0529 (15)	0.0477 (16)	0.0047 (14)	0.0093 (15)	−0.0105 (12)
C28	0.0600 (19)	0.096 (2)	0.076 (2)	0.0128 (16)	0.0248 (17)	0.0166 (17)
C21	0.0488 (15)	0.0629 (17)	0.0687 (19)	−0.0102 (13)	0.0030 (14)	−0.0025 (14)
C5	0.0465 (16)	0.0672 (18)	0.103 (2)	0.0012 (13)	0.0320 (16)	0.0114 (16)
C17	0.0462 (18)	0.083 (2)	0.108 (3)	−0.0003 (15)	0.0051 (18)	0.0060 (18)
C18	0.0473 (19)	0.120 (3)	0.115 (3)	−0.025 (2)	0.0028 (19)	−0.021 (2)
C27	0.0542 (15)	0.0337 (11)	0.0462 (15)	−0.0031 (11)	0.0098 (12)	0.0046 (10)
C19	0.063 (2)	0.082 (2)	0.073 (2)	−0.0371 (17)	0.0353 (17)	−0.0254 (17)
C7	0.0427 (13)	0.0379 (12)	0.0372 (13)	−0.0033 (10)	0.0081 (11)	0.0056 (10)
C29	0.0414 (13)	0.0289 (11)	0.0446 (14)	−0.0033 (10)	0.0022 (11)	0.0005 (10)
C32	0.0418 (14)	0.0489 (14)	0.0437 (15)	−0.0037 (11)	0.0024 (12)	−0.0056 (11)
C10	0.0432 (13)	0.0286 (10)	0.0336 (12)	−0.0042 (9)	0.0063 (10)	0.0032 (9)
C11	0.0457 (13)	0.0313 (11)	0.0358 (13)	−0.0061 (10)	0.0096 (10)	0.0047 (9)
C35	0.0574 (17)	0.0630 (16)	0.0658 (19)	−0.0037 (13)	0.0146 (15)	−0.0050 (14)
C22	0.0496 (14)	0.0313 (11)	0.0382 (13)	−0.0047 (10)	0.0017 (11)	0.0048 (10)
C3	0.0428 (14)	0.0458 (13)	0.0499 (16)	−0.0101 (11)	0.0035 (12)	0.0050 (11)
Cl1	0.0537 (4)	0.0854 (5)	0.1103 (7)	−0.0211 (4)	0.0320 (4)	0.0109 (5)

### Geometric parameters (Å, °)

**Table:**

S3—N2	1.6911 (18)	C1—C7	1.486 (3)
S3—O3	1.4202 (15)	C26—H26	0.9300
S3—O2	1.4328 (16)	C26—C25	1.385 (3)
S3—C29	1.746 (2)	C26—C27	1.375 (3)
S2—N2	1.7142 (17)	C31—H31	0.9300
S2—O4	1.4200 (18)	C31—C32	1.390 (3)
S2—O5	1.4218 (17)	C34—H34	0.9300
S2—C22	1.740 (2)	C34—C29	1.387 (3)
S1—C13	1.730 (2)	C23—H23	0.9300
S1—C11	1.736 (2)	C23—C24	1.370 (4)
Cl2—C3	1.729 (2)	C23—C22	1.384 (3)
C12—N2	1.436 (2)	C25—C24	1.378 (3)
C12—C13	1.371 (3)	C25—C28	1.507 (4)
C12—C10	1.428 (3)	C6—H6	0.9300
Cl3—C19	1.744 (3)	C6—C5	1.374 (3)
C2—H2	0.9300	C15—H15A	0.9700
C2—C1	1.389 (3)	C15—H15B	0.9700
C2—C3	1.379 (3)	C15—C16	1.505 (3)
N1—C7	1.339 (3)	C16—C21	1.372 (3)
N1—C11	1.337 (3)	C16—C17	1.373 (4)
C4—C5	1.369 (3)	C20—H20	0.9300
C4—C3	1.381 (3)	C20—C21	1.373 (3)
C4—Cl1	1.722 (2)	C20—C19	1.363 (4)
C9—H9	0.9300	C24—H24	0.9300
C9—C8	1.368 (3)	C28—H28C	0.9600
C9—C10	1.397 (3)	C28—H28B	0.9600
N3—H3	0.8600	C28—H28A	0.9600
N3—C14	1.329 (3)	C21—H21	0.9300
N3—C15	1.458 (3)	C5—H5	0.9300
C30—H30	0.9300	C17—H17	0.9300
C30—C31	1.366 (3)	C17—C18	1.387 (4)
C30—C29	1.382 (3)	C18—H18	0.9300
C33—H33	0.9300	C18—C19	1.356 (4)
C33—C34	1.363 (3)	C27—H27	0.9300
C33—C32	1.391 (3)	C27—C22	1.380 (3)
C8—H8	0.9300	C32—C35	1.495 (3)
C8—C7	1.402 (3)	C10—C11	1.395 (3)
C14—C13	1.489 (3)	C35—H35B	0.9600
C14—O1	1.217 (3)	C35—H35A	0.9600
C1—C6	1.385 (3)	C35—H35C	0.9600
			
S3—N2—S2	120.73 (9)	C25—C28—H28C	109.5
C12—N2—S3	119.03 (13)	C25—C28—H28B	109.5
C12—N2—S2	117.31 (13)	C25—C28—H28A	109.5
C12—C13—S1	111.82 (17)	O1—C14—N3	123.0 (2)
C12—C13—C14	133.09 (19)	O1—C14—C13	119.3 (2)
N2—S3—C29	107.55 (10)	C6—C1—C2	117.6 (2)
N2—S2—C22	104.92 (10)	C6—C1—C7	123.4 (2)
O3—S3—N2	105.25 (9)	C6—C5—H5	119.7
O3—S3—O2	120.47 (11)	C15—N3—H3	119.1
O3—S3—C29	110.55 (10)	H15A—C15—H15B	108.0
C2—C1—C7	119.0 (2)	C16—C15—H15A	109.4
C2—C3—Cl2	119.49 (19)	C16—C15—H15B	109.4
C2—C3—C4	120.4 (2)	C16—C21—C20	122.0 (3)
O4—S2—N2	107.64 (10)	C16—C21—H21	119.0
O4—S2—O5	120.70 (12)	C16—C17—H17	119.6
O4—S2—C22	110.32 (10)	C16—C17—C18	120.8 (3)
N1—C7—C8	121.7 (2)	C20—C21—H21	119.0
N1—C7—C1	115.83 (18)	C20—C19—Cl3	119.1 (3)
N1—C11—S1	122.14 (15)	C24—C23—H23	120.0
N1—C11—C10	126.0 (2)	C24—C23—C22	120.0 (2)
C4—C5—C6	120.6 (3)	C24—C25—C26	118.6 (2)
C4—C5—H5	119.7	C24—C25—C28	119.7 (3)
C4—C3—Cl2	120.05 (18)	H28C—C28—H28B	109.5
C9—C8—H8	119.5	H28C—C28—H28A	109.5
C9—C8—C7	120.9 (2)	H28B—C28—H28A	109.5
C9—C10—C12	132.10 (18)	C21—C16—C15	121.5 (2)
N3—C14—C13	117.6 (2)	C21—C16—C17	117.9 (2)
N3—C15—H15A	109.4	C21—C20—H20	120.7
N3—C15—H15B	109.4	C5—C4—C3	119.0 (2)
N3—C15—C16	111.3 (2)	C5—C4—Cl1	119.0 (2)
C30—C31—H31	119.3	C5—C6—C1	121.4 (2)
C30—C31—C32	121.3 (2)	C5—C6—H6	119.3
C30—C29—S3	120.35 (17)	C17—C16—C15	120.6 (2)
C30—C29—C34	119.7 (2)	C17—C18—H18	120.4
C33—C34—H34	120.4	C18—C17—H17	119.6
C33—C34—C29	119.3 (2)	C18—C19—Cl3	119.5 (3)
C33—C32—C35	120.8 (2)	C18—C19—C20	121.4 (3)
C8—C9—H9	120.8	C27—C26—H26	119.3
C8—C9—C10	118.39 (19)	C27—C26—C25	121.3 (2)
C8—C7—C1	122.5 (2)	C27—C22—S2	121.61 (18)
O2—S3—N2	104.19 (9)	C27—C22—C23	120.0 (2)
O2—S3—C29	107.97 (11)	C19—C20—H20	120.7
C14—N3—H3	119.1	C19—C20—C21	118.6 (3)
C14—N3—C15	121.7 (2)	C19—C18—C17	119.2 (3)
C14—C13—S1	114.78 (15)	C19—C18—H18	120.4
C1—C2—H2	119.5	C7—C8—H8	119.5
C1—C6—H6	119.3	C29—C30—H30	119.9
C26—C25—C28	121.7 (2)	C29—C34—H34	120.4
C26—C27—H27	120.4	C32—C33—H33	118.8
C26—C27—C22	119.2 (2)	C32—C31—H31	119.3
C13—S1—C11	91.48 (10)	C32—C35—H35B	109.5
C13—C12—N2	123.7 (2)	C32—C35—H35A	109.5
C13—C12—C10	113.50 (17)	C32—C35—H35C	109.5
C31—C30—H30	119.9	C10—C12—N2	122.67 (18)
C31—C30—C29	120.2 (2)	C10—C9—H9	120.8
C31—C32—C33	117.2 (2)	C10—C11—S1	111.85 (16)
C31—C32—C35	122.0 (2)	C11—N1—C7	116.45 (17)
C34—C33—H33	118.8	C11—C10—C12	111.33 (18)
C34—C33—C32	122.3 (2)	C11—C10—C9	116.54 (19)
C34—C29—S3	119.99 (18)	H35B—C35—H35A	109.5
C23—C24—C25	120.8 (2)	H35B—C35—H35C	109.5
C23—C24—H24	119.6	H35A—C35—H35C	109.5
C23—C22—S2	118.28 (17)	C22—C23—H23	120.0
O5—S2—N2	103.82 (9)	C22—C27—H27	120.4
O5—S2—C22	108.17 (12)	C3—C2—H2	119.5
C25—C26—H26	119.3	C3—C2—C1	120.9 (2)
C25—C24—H24	119.6	C3—C4—Cl1	121.94 (19)
			
C12—C10—C11—S1	−1.8 (2)	C34—C33—C32—C35	−179.3 (2)
C12—C10—C11—N1	−179.6 (2)	O5—S2—N2—S3	−160.20 (13)
N2—S3—C29—C30	86.4 (2)	O5—S2—N2—C12	0.2 (2)
N2—S3—C29—C34	−94.24 (19)	O5—S2—C22—C23	−32.3 (2)
N2—S2—C22—C23	78.0 (2)	O5—S2—C22—C27	144.55 (18)
N2—S2—C22—C27	−105.11 (18)	C25—C26—C27—C22	−0.6 (3)
N2—C12—C13—S1	−177.84 (16)	O1—C14—C13—S1	35.1 (3)
N2—C12—C13—C14	−4.7 (4)	O1—C14—C13—C12	−137.8 (3)
N2—C12—C10—C9	0.7 (4)	C6—C1—C7—N1	176.8 (2)
N2—C12—C10—C11	178.63 (18)	C6—C1—C7—C8	−2.8 (3)
O3—S3—N2—S2	−32.14 (15)	C15—N3—C14—C13	−177.6 (2)
O3—S3—N2—C12	167.77 (16)	C15—N3—C14—O1	3.3 (4)
O3—S3—C29—C30	−159.17 (18)	C15—C16—C21—C20	178.9 (3)
O3—S3—C29—C34	20.2 (2)	C15—C16—C17—C18	−179.4 (3)
C2—C1—C6—C5	1.5 (4)	C16—C17—C18—C19	0.9 (5)
C2—C1—C7—N1	−0.7 (3)	C24—C23—C22—S2	175.52 (19)
C2—C1—C7—C8	179.8 (2)	C24—C23—C22—C27	−1.4 (4)
O4—S2—N2—S3	−31.16 (16)	C28—C25—C24—C23	−177.6 (2)
O4—S2—N2—C12	129.26 (16)	C21—C16—C17—C18	1.4 (5)
O4—S2—C22—C23	−166.28 (18)	C21—C20—C19—Cl3	−176.6 (2)
O4—S2—C22—C27	10.6 (2)	C21—C20—C19—C18	2.3 (5)
C9—C8—C7—N1	−1.1 (3)	C5—C4—C3—Cl2	−179.9 (2)
C9—C8—C7—C1	178.5 (2)	C5—C4—C3—C2	2.3 (4)
C9—C10—C11—S1	176.53 (16)	C17—C16—C21—C20	−2.0 (4)
C9—C10—C11—N1	−1.3 (3)	C17—C18—C19—Cl3	176.1 (3)
N3—C14—C13—S1	−144.01 (18)	C17—C18—C19—C20	−2.9 (5)
N3—C14—C13—C12	43.0 (4)	C27—C26—C25—C24	−1.1 (4)
N3—C15—C16—C21	−48.3 (3)	C27—C26—C25—C28	178.1 (2)
N3—C15—C16—C17	132.5 (3)	C19—C20—C21—C16	0.1 (4)
C30—C31—C32—C33	−0.5 (3)	C7—N1—C11—S1	−176.33 (16)
C30—C31—C32—C35	−179.8 (2)	C7—N1—C11—C10	1.3 (3)
C33—C34—C29—S3	−179.88 (19)	C7—C1—C6—C5	−176.0 (2)
C33—C34—C29—C30	−0.5 (3)	C29—S3—N2—S2	85.76 (14)
C8—C9—C10—C12	177.9 (2)	C29—S3—N2—C12	−74.33 (18)
C8—C9—C10—C11	0.1 (3)	C29—C30—C31—C32	−0.9 (4)
O2—S3—N2—S2	−159.80 (12)	C32—C33—C34—C29	−0.9 (4)
O2—S3—N2—C12	40.11 (19)	C10—C12—N2—S3	91.2 (2)
O2—S3—C29—C30	−25.5 (2)	C10—C12—N2—S2	−69.5 (2)
O2—S3—C29—C34	153.87 (18)	C10—C12—C13—S1	−1.1 (2)
C14—N3—C15—C16	161.2 (2)	C10—C12—C13—C14	172.0 (2)
C1—C2—C3—Cl2	−177.56 (18)	C10—C9—C8—C7	1.0 (3)
C1—C2—C3—C4	0.2 (4)	C11—S1—C13—C12	0.07 (17)
C1—C6—C5—C4	1.0 (4)	C11—S1—C13—C14	−174.39 (18)
C26—C25—C24—C23	1.6 (4)	C11—N1—C7—C8	−0.1 (3)
C26—C27—C22—S2	−174.91 (18)	C11—N1—C7—C1	−179.62 (19)
C26—C27—C22—C23	1.9 (3)	C22—S2—N2—S3	86.35 (14)
C13—S1—C11—N1	178.89 (19)	C22—S2—N2—C12	−113.23 (17)
C13—S1—C11—C10	0.99 (17)	C22—C23—C24—C25	−0.4 (4)
C13—C12—N2—S3	−92.3 (2)	C3—C2—C1—C6	−2.1 (3)
C13—C12—N2—S2	106.9 (2)	C3—C2—C1—C7	175.5 (2)
C13—C12—C10—C9	−176.1 (2)	C3—C4—C5—C6	−2.9 (4)
C13—C12—C10—C11	1.8 (3)	Cl1—C4—C5—C6	175.7 (2)
C31—C30—C29—S3	−179.22 (18)	Cl1—C4—C3—Cl2	1.5 (3)
C31—C30—C29—C34	1.4 (3)	Cl1—C4—C3—C2	−176.26 (18)
C34—C33—C32—C31	1.4 (4)		

### Hydrogen-bond geometry (Å, °)

**Table:**

*D*—H···*A*	*D*—H	H···*A*	*D*···*A*	*D*—H···*A*
N3—H3···O2	0.86	2.11	2.945 (3)	162
C34—H34···S1^i^	0.93	2.79	3.714 (2)	172

Symmetry codes: (i) *x*, *y*+1, *z*.
